# Supplementary figures and images for: The p53 protein is a suppressor of Atox1 copper chaperon in tumor cells under genotoxic effects
Source: PLoS One. 2023 Dec 21;18(12):e0295944. doi: 10.1371/journal.pone.0295944 (PMC10735018; doi:10.1371/journal.pone.0295944)

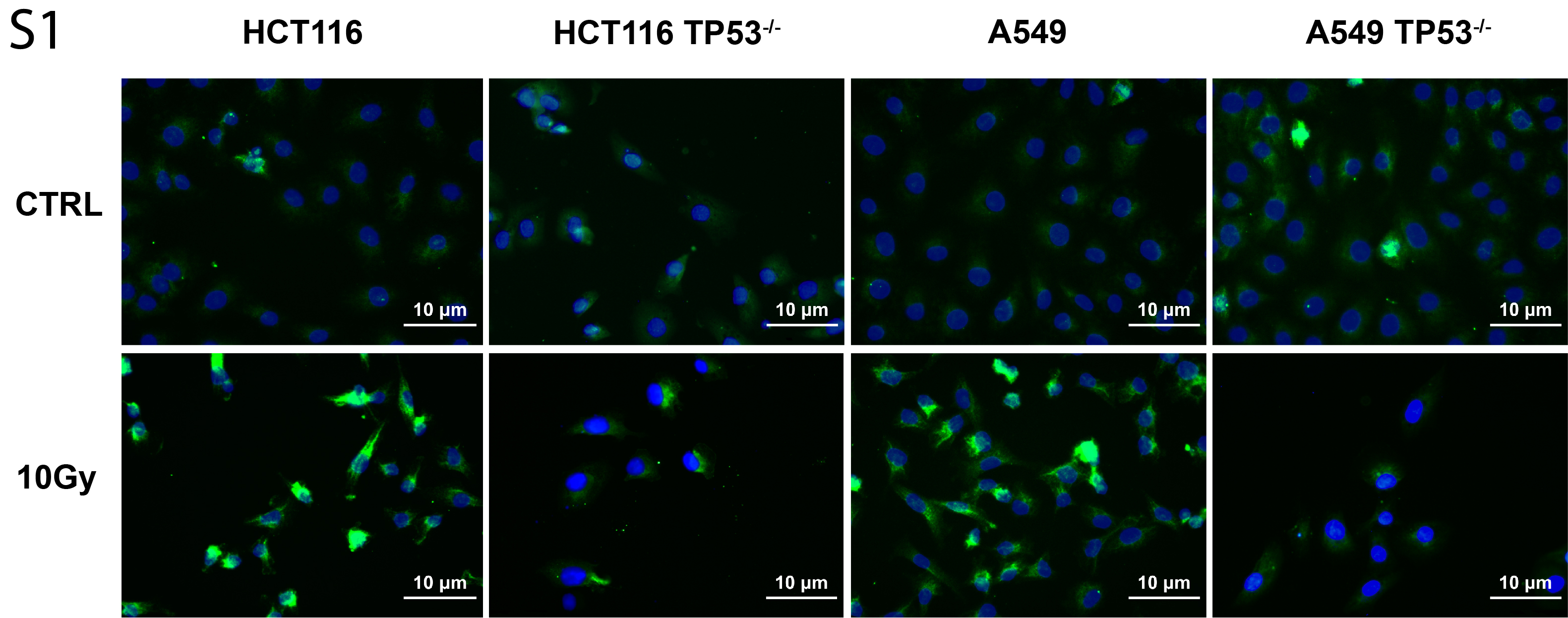

Supplement: S1 Fig — Immunofluorescence staining with primary antibodies to Atox1 and secondary antibodies with AlexaFluor488. DAPI was used for nuclei staining. TP53-/-–cells without TP53. For all experiments: n = 3, mean +/− SEM, two-way ANOVA, p < 0.05. (TIFF) [file pone.0295944.s001.tiff]

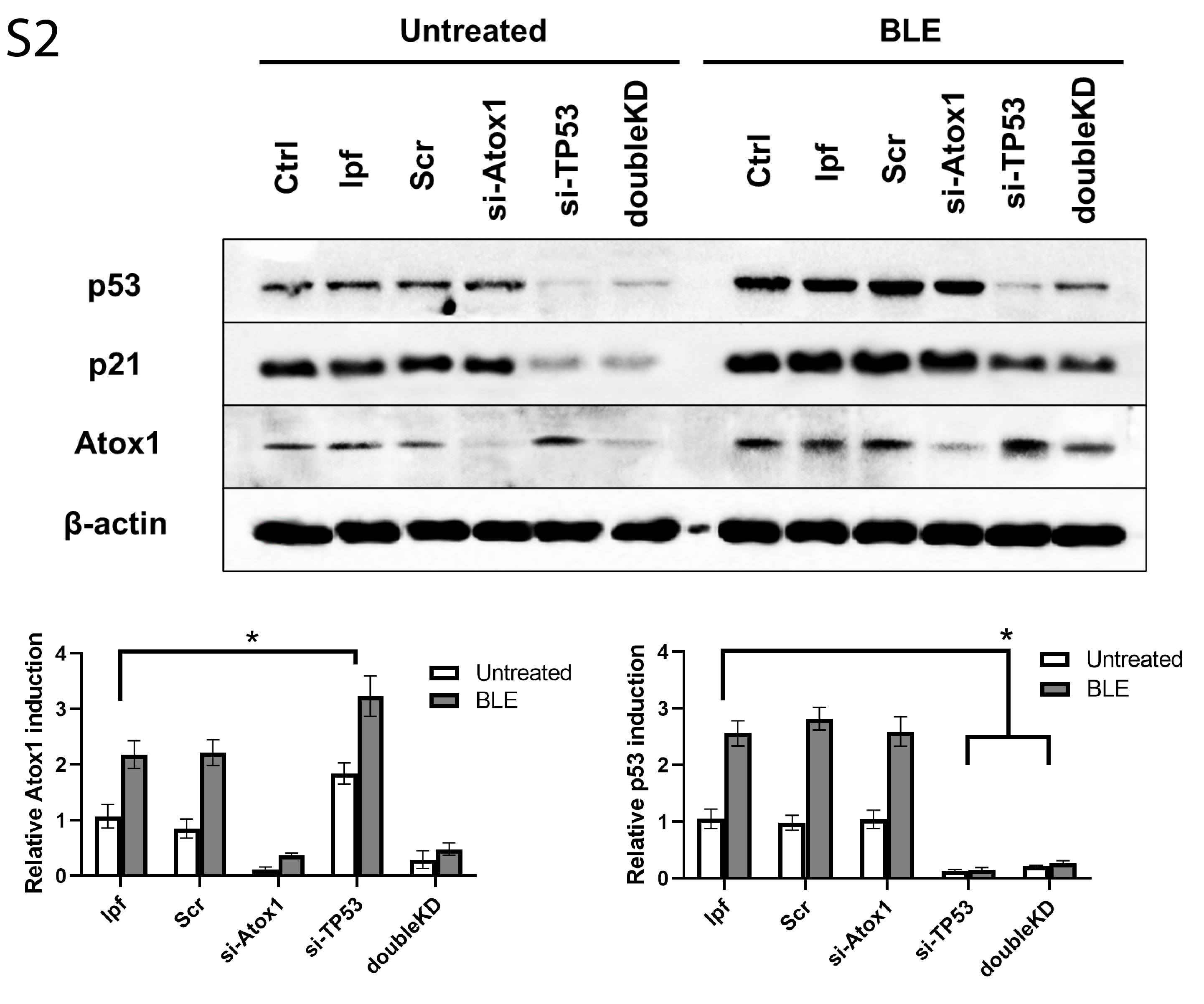

Supplement: S2 Fig — Immunoblotting with antibodies to p53, p21, and Atox1; beta-actin was used as a normalization. ATOX1 (si-ATOX1), TP53 (si-TP53) or double ATOX1/ TP53 (doubleKD) knockdowns were used in the absence (Untreated) and presence (10μM BLE) of bleomycin, 24h after exposure. (TIFF) [file pone.0295944.s002.tiff]
