# Supplementary material for: The p53 protein is a suppressor of Atox1 copper chaperon in tumor cells under genotoxic effects
Source: PLoS One. 2023 Dec 21;18(12):e0295944. doi: 10.1371/journal.pone.0295944 (PMC10735018; doi:10.1371/journal.pone.0295944)
Supplement: S1 Raw images — (PDF) [file pone.0295944.s003.pdf]

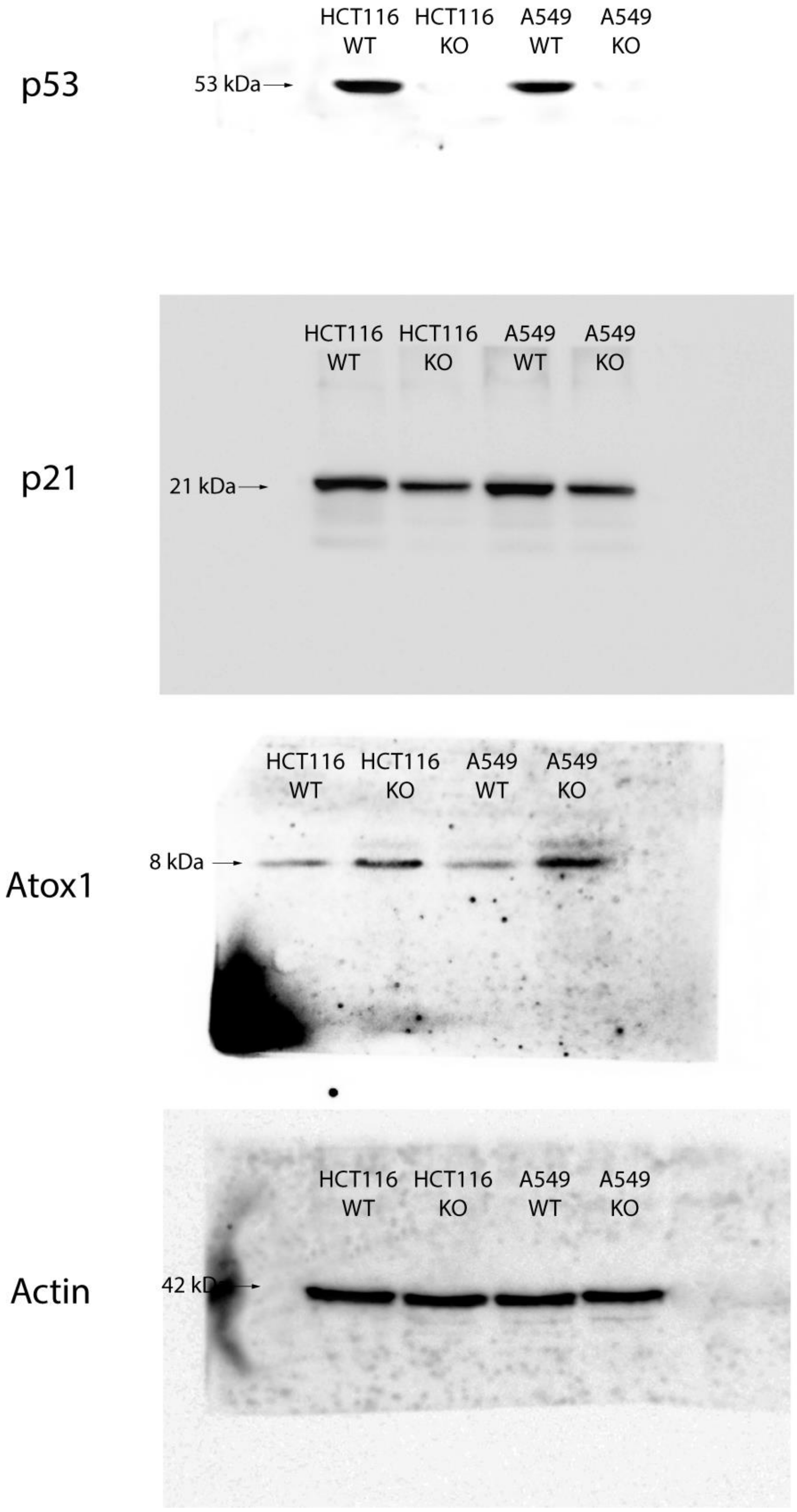

Raw images of western blot presented on Figure 1A.

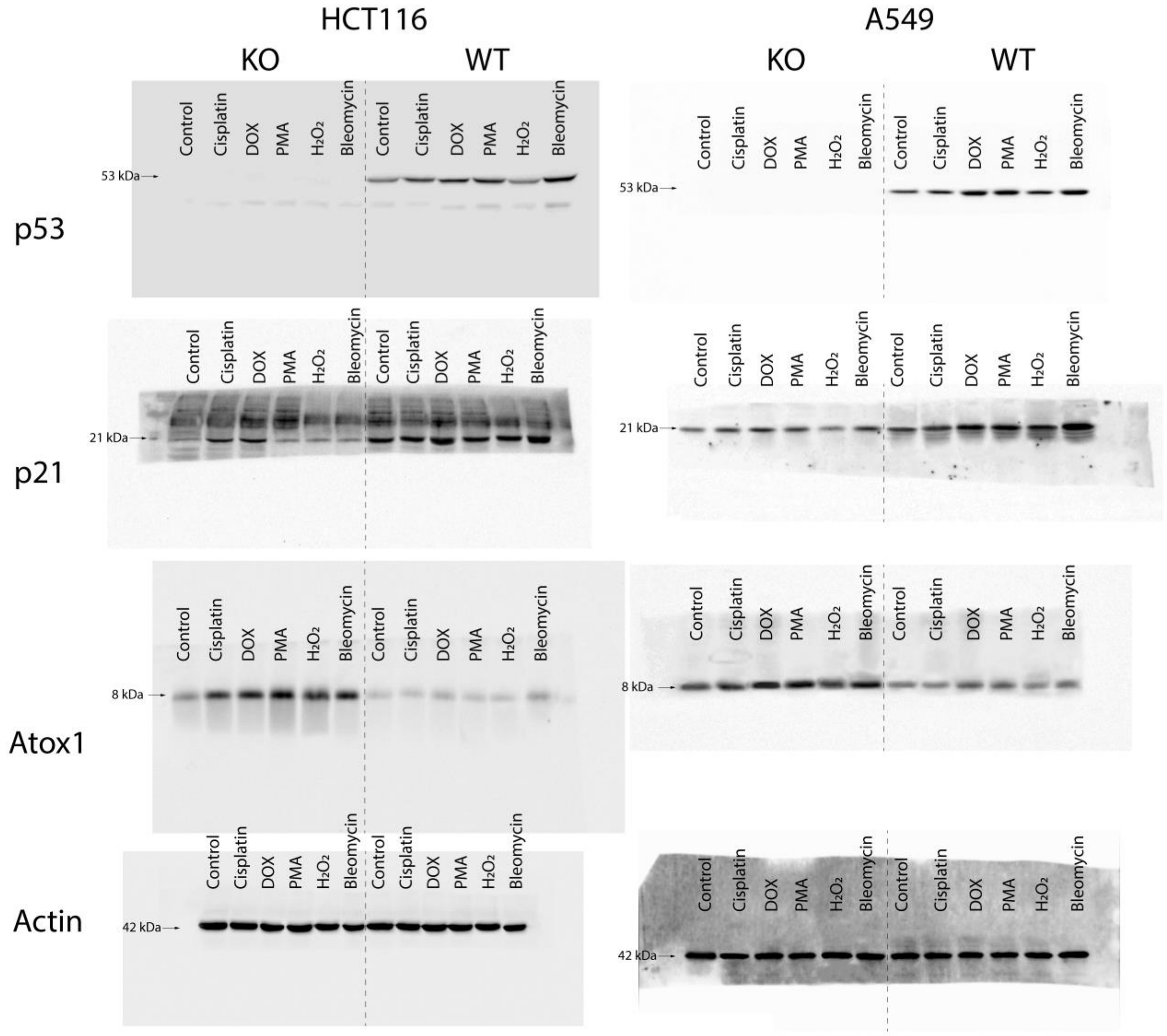

Raw images of western blot presented on Figure 2A.

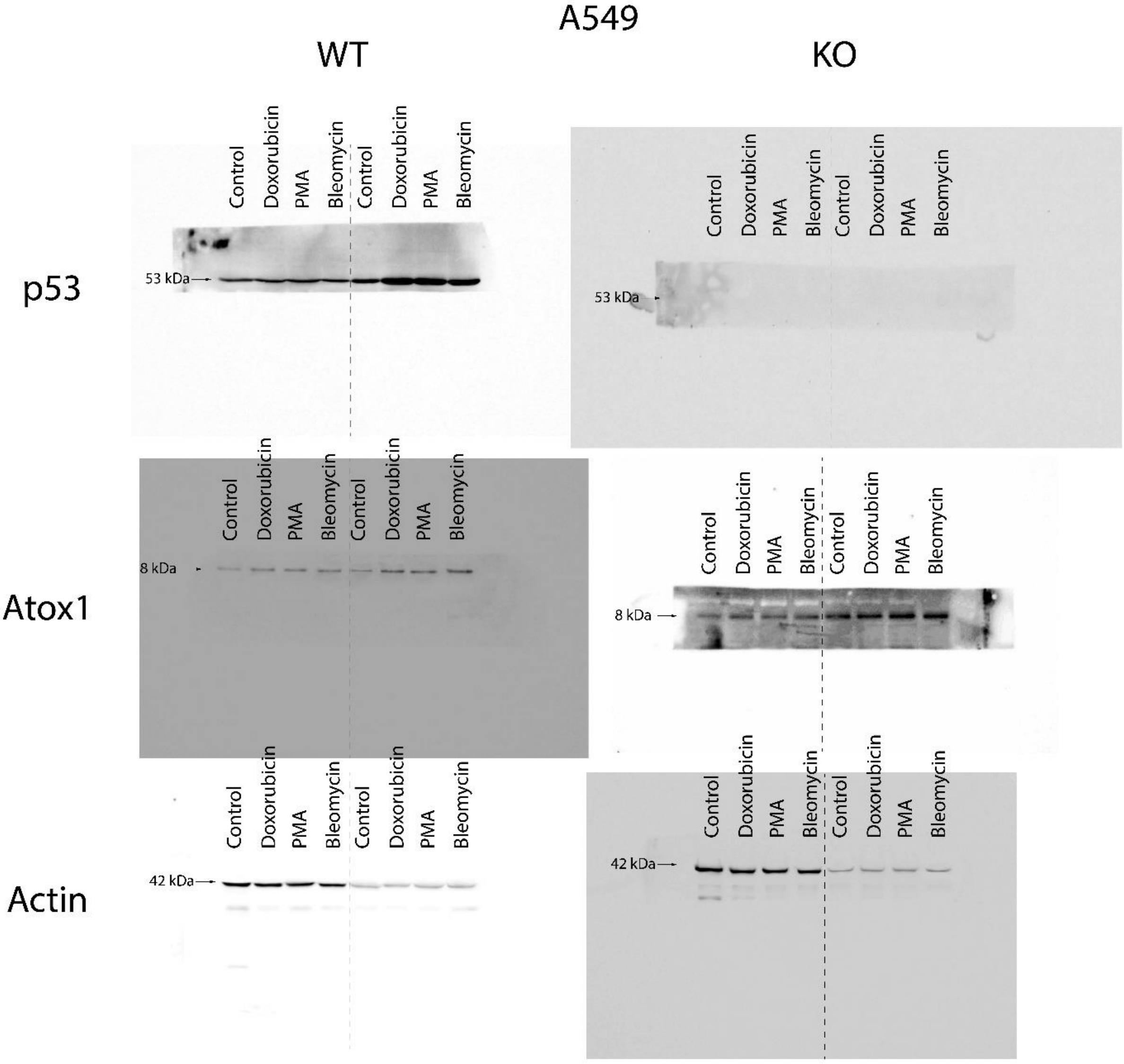

Raw images of western blot presented on Figure 3B.

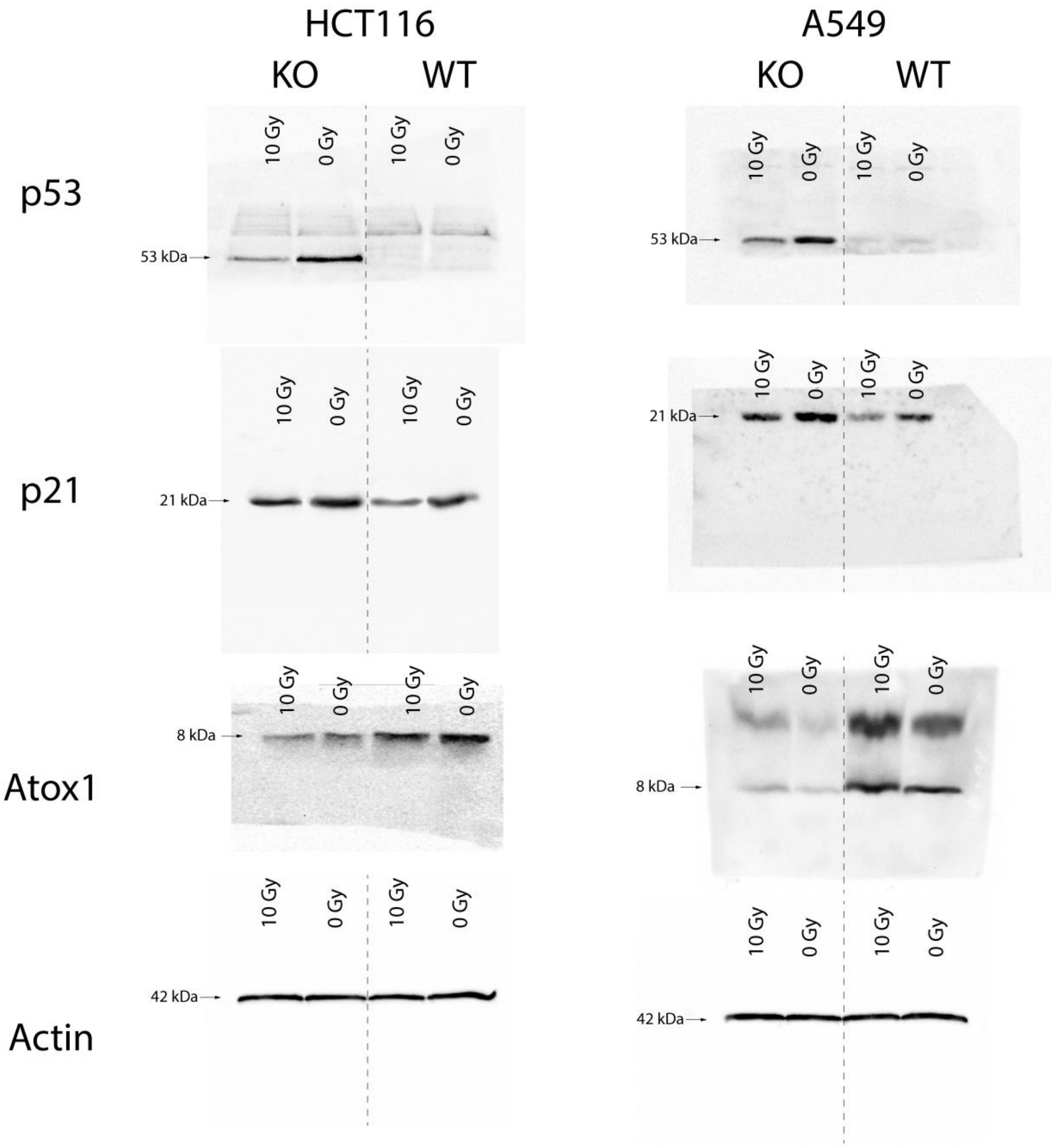

Raw images of western blot presented on Figure 4B.

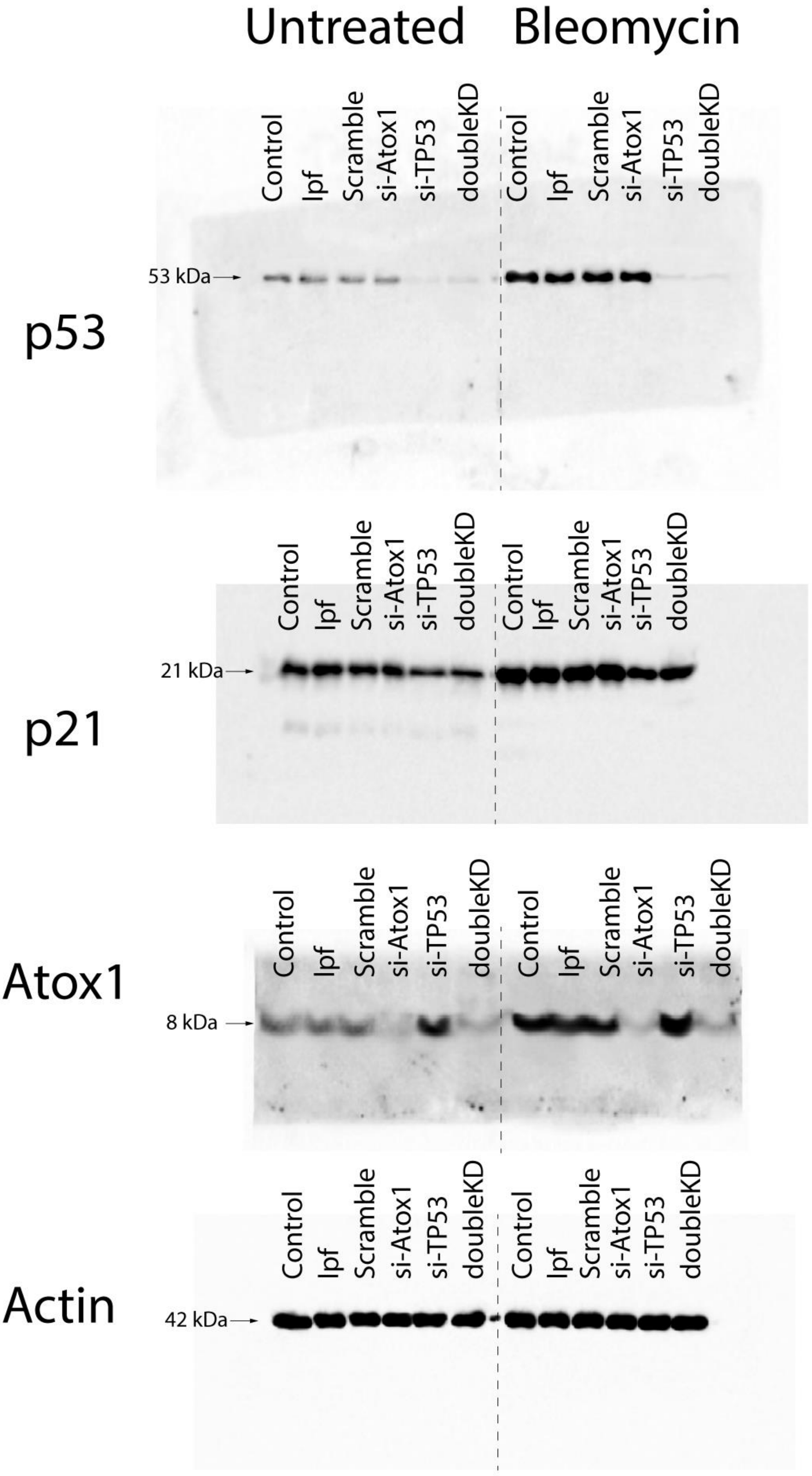

Raw images of western blot presented on Figure 5B.
